# Challenges and lessons from a vector control campaign targeting *Glossina palpalis palpalis* in an isolated protected forest area in Abidjan, Côte d’Ivoire

**DOI:** 10.1051/parasite/2025017

**Published:** 2025-04-15

**Authors:** Yao Jean Rodrigue Konan, Bi Tra Dieudonné Ta, Djakaridja Berté, Bamoro Coulibaly, Kinifo Donatien Coulibaly, Nick Steven Egnankon, Foungniguée Diarrassouba, Kouassi Albert Djabo, Stéphanie Watier-Grillot, Jean-Paul Demoncheaux, Koffi Alain De Marie Kouadio, Louis N’Dri, Philippe Solano, Sophie Ravel, Guy Pacôme Adingra, Antoine Barreaux, Adeline Ségard, Dramane Kaba, Vincent Jamonneau, Thierry De Meeûs, Vincent Djohan

**Affiliations:** 1 Institut Pierre Richet, Institut National de Sante Publique 01 BP 1500 Bouaké Côte d’Ivoire; 2 Université Felix Houphouët-Boigny 01 BPV 34 Abidjan Côte d’Ivoire; 3 Université Peleforo Gon Coulibaly BP 1328 Korhogo Côte d’Ivoire; 4 Université Jean Lorougnon Guédé BP 150 Daloa Côte d’Ivoire; 5 Direction interarmées du service de santé pour l’Afrique Centrale et de l’Ouest BP 175 Abidjan Côte d’Ivoire; 6 Intertryp, Université de Montpellier, Cirad, IRD, TA A-17/G, Campus International de Baillarguet 34398 Montpellier Cedex 5 France; 7 Animal health Theme, International Centre of Insect Physiology and Ecology (ICIPE) PO Box 30772-00100 Nairobi Kenya

**Keywords:** Tsetse flies, Vector control, Tiny target, African trypanosomoses, Population genetics, Resistance

## Abstract

Vector control (VC) is one of the strategies employed to manage African trypanosomoses. This study aimed at assessing the effectiveness of a VC campaign against *Glossina palpalis palpalis* using tiny targets (TTs) impregnated with insecticide in an isolated, protected forest in Abidjan, Côte d’Ivoire, while considering ecological, genetic, and operational factors. Between January 2020 and September 2022, 2,712 TTs were deployed at 684 sites, covering a total area of 1.7 km^2^. VC monitoring was conducted using Vavoua traps during 12 evaluation surveys, between June 2020 and March 2023. Five months after the initial TT deployment, tsetse fly density had decreased by 98.53%. Although tsetse density remained low due to TT redeployment and reinforcement, there was a significant increase a few months after the last redeployment. VC appeared to have minimal impact on the genetic structuring of *G. p. palpalis*. This suggested recruitment of local surviving tsetse flies all along the VC campaign due to a low probability of tsetse coming into contact with TTs, or to the evolution of behavioral or physiological resistance to control efforts. The genetic study revealed that one of the microsatellite markers used, the GPCAG locus, exhibited a selection signature possibly in response to VC. This could partly explain the challenges encountered in eliminating a seemingly isolated tsetse population thriving in a particularly favorable habitat.

## Abstract (fr)

La lutte antivectorielle (LAV) fait partie des stratégies de contrôle des trypanosomoses africaines. La présente étude visait à évaluer l’efficacité d’une campagne de LAV contre *Glossina palpalis palpalis* avec des écrans de type « tiny targets » (TT) imprégnés d’insecticide dans une forêt isolée et protégée à Abidjan, Côte d’Ivoire, en tenant compte des facteurs écologiques, génétiques et opérationnels. Entre janvier 2020 et septembre 2022, 2 712 TT ont été déployés sur 684 sites totalisant une superficie de 1,7 km^2^. Le suivi de la LAV a été effectué en utilisant des pièges Vavoua lors de 12 enquêtes d’évaluation menées entre juin 2020 et mars 2023. Cinq mois après le premier déploiement des TT, la densité de glossines avait chuté de 98,53 %. Elle est restée faible grâce aux redéploiements et renforcements en TT, mais a ré-augmenté significativement quelques mois après le dernier redéploiement. Elle semble avoir peu impacté la structuration génétique de *G. p. palpalis*. Cela suggère un recrutement local de mouches survivantes tout au long de la campagne de LAV, dû à une faible probabilité pour les glossines de rencontrer un TT ou à une possible stratégie d’évitement ou de résistance aux efforts de lutte. L’étude génétique a d’ailleurs révélé que l’un des marqueurs microsatellite utilisé, le locus GPCAG, présentait une signature de sélection possiblement en réponse à la LAV. Ceci permettrait en partie d’expliquer les difficultés rencontrées pour éliminer une population de glossines apparemment isolée mais occupant un biotope particulièrement favorable.

## Introduction

*Glossina palpalis palpalis* is the main vector of *Trypanosoma brucei gambiense* responsible for human African trypanosomiasis (HAT) in West and Central Africa [8]. It is also one of the major vectors of animal African trypanosomiasis (AAT), caused by *T. b. brucei, T. congolense* and *T. vivax* [26] in the southern part of Côte d’Ivoire [35, 49, 59]. In Côte d’Ivoire, HAT was eliminated as a public health problem in 2020 [40, 61]. However, *G. p. palpalis* is still a risk factor for the re-emergence of this disease due to the existence of residual human or animal reservoirs of *T. b. gambiense* [7, 37, 41, 44, 45, 73]. In Côte d’Ivoire, AAT probably occurs throughout the distribution range of *G. p. palpalis* and infections have recently been reported in the Central-West and South-West of the country, with high prevalence of *T. b. brucei* and *T. congolense* in pigs [57, 73].

The military base of the French Armed Forces in Côte d’Ivoire, which is located in Abidjan, includes a forest area where *G. p. palpalis* has been identified as a vector of AAT responsible for the death of military working dogs (MWDs) [9, 43, 47, 78]. A recent study was carried out on the ecology of tsetse flies in this conserved and protected urban forest area [46]. Two entomological surveys conducted in May 2019 (rainy season) and January 2020 (dry season) confirmed the presence of *G. p. palpalis* inside the French military base. The highest densities were observed in the southern zone, which is a particularly favorable biotope, both because of the humidity and vegetation present, but also because of the presence of wild animals (bushbucks, monitor lizards, civets and crocodiles) on which tsetse flies can feed. Three tsetse flies were also captured in a peripheral gardening area outside the base. The type of vegetation and the extensive use of insecticides in this area seemed to indicate that these tsetse flies came from inside the French military base, but that they could go outside, in particular to feed on available hosts. However, this hypothesis remained to be verified. In addition, the question of the risk of exchanges of tsetse between the base and more distant neighboring areas remained. These results highlighted not only a risk of animal trypanosomes transmission, but also of possible *T. b. gambiense* transmission. Importantly, the French soldiers and support staff who stay at the base regularly carry out field missions or military exercises in HAT foci in Côte d’Ivoire and other endemic countries where they can potentially be infected [46]. The authorities of the French Armed Forces in Côte d’Ivoire therefore decided to implement a vector control (VC) campaign to protect human and MWDs at the military base.

VC is a key part of the integrated control of African trypanosomoses [6]. In the past, control campaigns against *G. p. palpalis* were based on the deployment of insecticide-impregnated monoconical and biconical traps [49, 50]. More recently, a new and less costly control method has been developed using tiny targets (TTs) impregnated with deltamethrin [52, 66, 67, 71]. This method has proved highly effective in VC campaigns to interrupt HAT transmission, notably against *G. fuscipes fuscipes* in Chad [52] and Uganda [71], *G. fuscipes quanzensis* in the Democratic Republic of Congo [72], *G. p. gambiensis* in Guinea [58] and in particular against *G. p. palpalis* in the HAT focus in Bonon in Central-West Côte d’Ivoire [39].

Following these successes, TTs were used for the VC campaign implemented at the aforementioned French military base. They were particularly well suited to the environment composed of both anthropized and preserved areas, and where other tsetse control methods such as biocidal products spraying, including pour-on, are undesirable. The aim was to reduce the risk of trypanosome transmission, with the hope of definitively interrupting their transmission cycle by using only this method.

This article describes (i) entomological surveys carried out around the French military base in Abidjan to assess the risk of tsetse fly introduction from a neighboring area, (ii) the VC strategy implemented in the study area, (iii) methods for assessing the impact of VC on entomological parameters in order to refine the deployment strategy for TTs, (iv) a population genetics approach aimed at assessing the impact of VC on the biology of this population, and (v) the difficulties encountered in controlling an apparently isolated tsetse population occupying a particularly favorable biotope. Sharing this experience of a VC campaign provides insights for future interventions, especially in small areas of intervention.

## Materials and methods

### Study area

**Figure 1:**
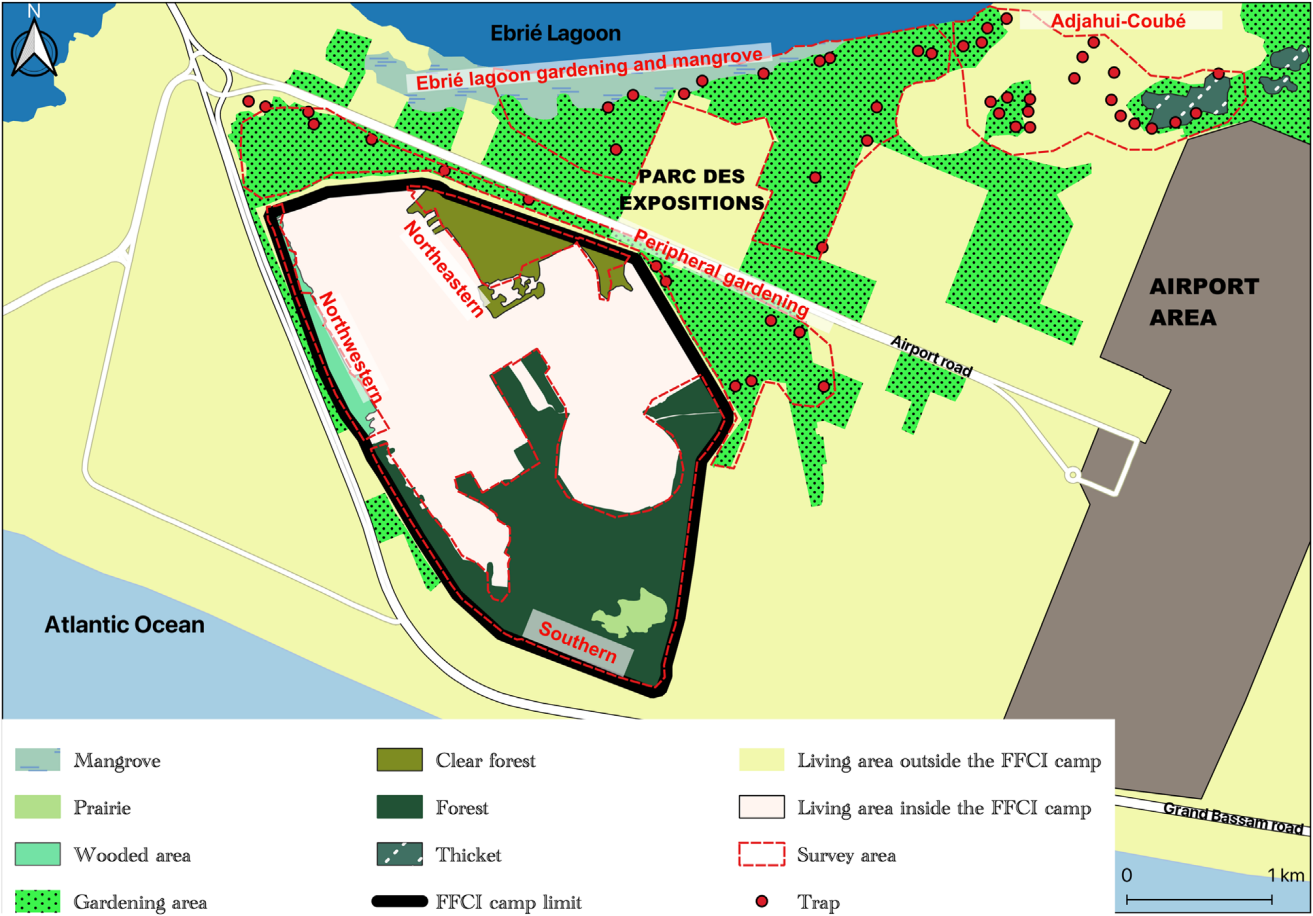
Study area (Source: Institut Pierre Richet, 2024). Figure adapted from Figure 1 of Konan [46]. It represents (red boundary) the three zones of the French military base in which the VC campaign was conducted and the three zones in which traps (in red) were set as part of the entomological surveys conducted outside the base.

Covering a total surface area of 1.7 km^2^, the French military base covered by this study is located South of Abidjan, within the commune of Port-Bouët, bordered by the Atlantic Ocean, the Ebrié Lagoon, and the Félix Houphouët-Boigny international airport (Fig. 1). It comprises both living spaces and a non-urbanized area, the latter harboring a biotope potentially conducive to tsetse flies. In a previous study [46], this non-urbanized area was divided into three zones based on the vegetation present: (i) the southern zone, characterized by dense evergreen forest encompassing a meadow where green waste is incinerated (referred to as the burning zone), (ii) the northeastern zone, situated near a kennel, featuring a light forest with open undergrowth, and (iii) the northwestern zone, wooded yet lacking undergrowth and partially marshy, positioned between the base boundary and the living space. A fourth zone consisting in a market gardening area on the northern outskirts of the base (lying between the base boundary and the airport road) has been also identified as potentially conducive to tsetse flies (peripheral gardening area) (Fig. 1). In the framework of the present study, an analysis based on satellite images and field surveys identified two other sites potentially favorable to tsetse flies to the north of the base between the airport road and the Ebrié lagoon: an area of market gardening and mangrove swamps along the Ebrié lagoon (Ebrié lagoon gardening and mangrove area) and an area of groves near the village of Adjahui-Coubé (Adjahui-Coubé area) (Fig. 1).

### Entomological surveys carried out around the French military base

An entomological survey was carried out in June 2020 using 14 Vavoua traps [48] in the peripheral gardening area and 14 Vavoua traps in the Ebrié lagoon gardening and mangrove area. The traps were set for four consecutive days with tsetse fly collection every 24 h. In September 2020, an entomological survey was carried out using 22 Vavoua traps in the Adjahui-Coubé area. The traps were set for 2 consecutive days with tsetse fly collection every 24 h. The distribution of these 50 traps in the affected areas is given in Figure 1.

### Control strategies

The VC strategy inside the French military base consisted of deploying TTs impregnated with deltamethrin [66] for 3 years (2020–2022) in biotopes favorable to tsetse flies identified during the baseline entomological surveys in May 2019 (rainy season T_−1_) and January 2020 (dry season T_0_) [46]. The first deployment was carried out in January 2020 just after the T_0_ baseline entomological survey. All the TTs installed were systematically replaced every 6 months to limit a possible reduction in effectiveness due to leaching by the high rainfall observed in the area and the breakdown of the active ingredient due to the UV exposure. The control strategy consisted of adapting the deployments, considering the results of previous entomological evaluations. Additional TTs were added during the campaign to reinforce control in areas where tsetse flies were being captured.

**Figure 2:**
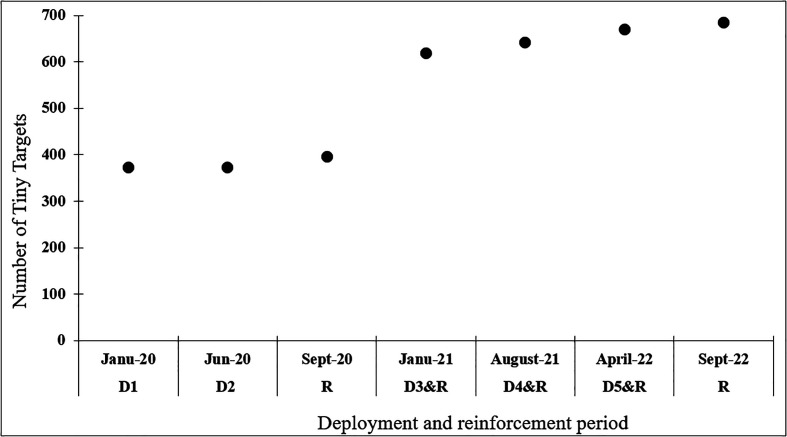
Deployments and reinforcements of tiny targets during the vector control campaign. DX: Deployment; D1: First deployment; D2 to D5: Replacement of all previously set tiny targets; R: Reinforcement with additional tiny targets.

**Figure 3:**
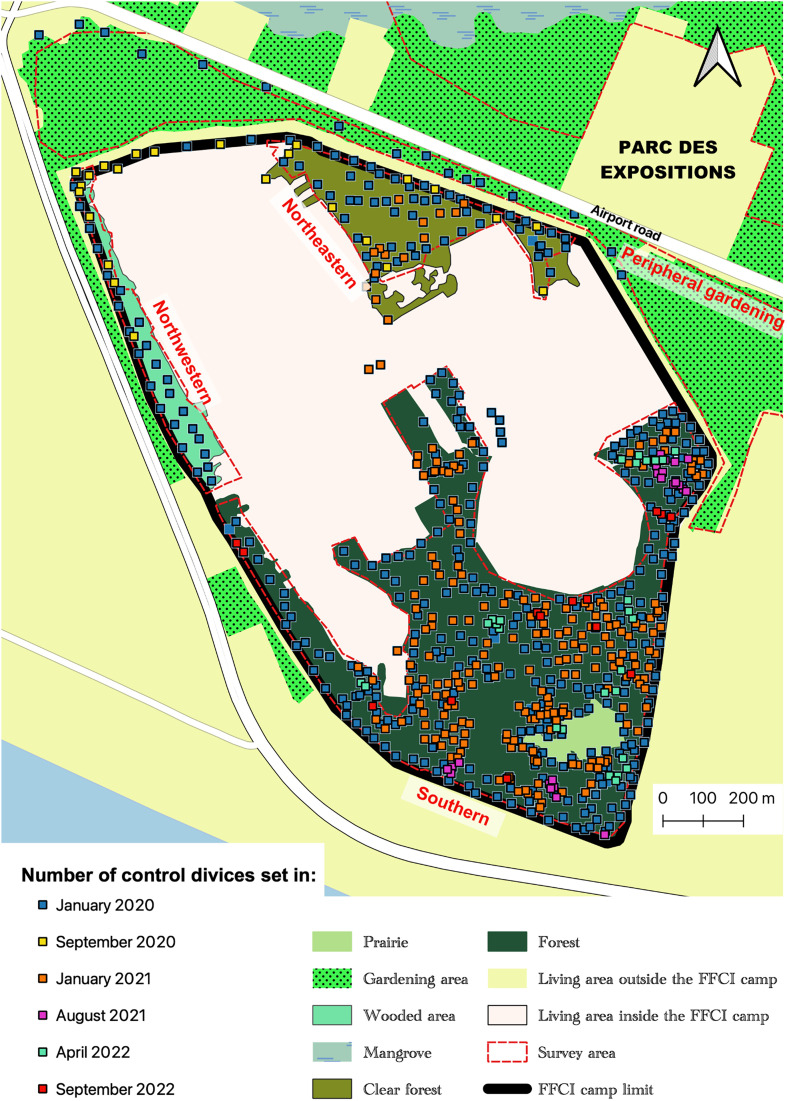
Distribution of control devices in the study area in September 2022 (last reinforcements) (Source: Institut Pierre Richet, 2024).

The evolution of the number of TTs deployed or redeployed (D) throughout the VC campaign is described in Figure 2. The first deployment (D1) in January 2020 consisted of installing 372 TTs, *i.e.* 219 TTs/km^2^. In June 2020 (D2) all the TTs were replaced. The three other deployments (D3 January 2021, D4 August 2021 and D5 April 2022) consisted of renewing the TT previously installed, including those installed to reinforce control at sites where tsetse fly had been captured during entomological evaluations (see below). A total of 312 additional TTs were installed during five reinforcements (R): 24 in September 2020, 222 in January 2021, 24 in August 2021, 27 in April 2022, and 15 in September 2022 (Fig. 2). This meant a total of 684 TTs deployed in September 2022 in the study area (Fig. 3), *i.e.*, 400 TTs/km^2^.

### Monitoring of VC

All the evaluations carried out to measure the impact of VC on the density and distribution of tsetse flies were carried out using unimpregnated Vavoua traps [48]. They were set at selected sites for four consecutive days, with tsetse being collected every 24 h. A total of 12 evaluations (T_1_–T_12_) were carried out. Quarterly evaluations were planned, but the first one (T_1_) was carried out in June 2020, 5 months after the first deployment, due to the temporary inaccessibility of the area as a result of the COVID-19 pandemic. The 11 other evaluations were then carried out at regular intervals of around 3 months. The last evaluation (T_12_) was carried out in March 2023 (Table 1). The results of the evaluations were compared with those obtained during the previously published T_0_ baseline survey [46] also described in Supplementary material 1.

**Table 1:** Number and status of traps set for each evaluation.

Evaluation	Period	T_0_ sentinel traps	T_0_ other traps	Supplementary traps	Total traps
T_1_	June 2020	25	ND	ND	25
T_2_	September 2020	25	ND	ND	25
T_3_	December 2020	25	ND	ND	25
T_4_	March 2021	25	55	ND	80
T_5_	June 2021	25	ND	ND	25
T_6_	October 2021	25	ND	ND	25
T_7_	December 2021	25	ND	ND	25
T_8_	March 2022	25	55	27	107
T_9_	June 2022	25	55	27	107
T_10_	September 2022	25	55	27	107
T_11_	December 2022	25	55	27	107
T_12_	March 2023	25	55	27	107

ND: Not defined.

**Figure 4:**
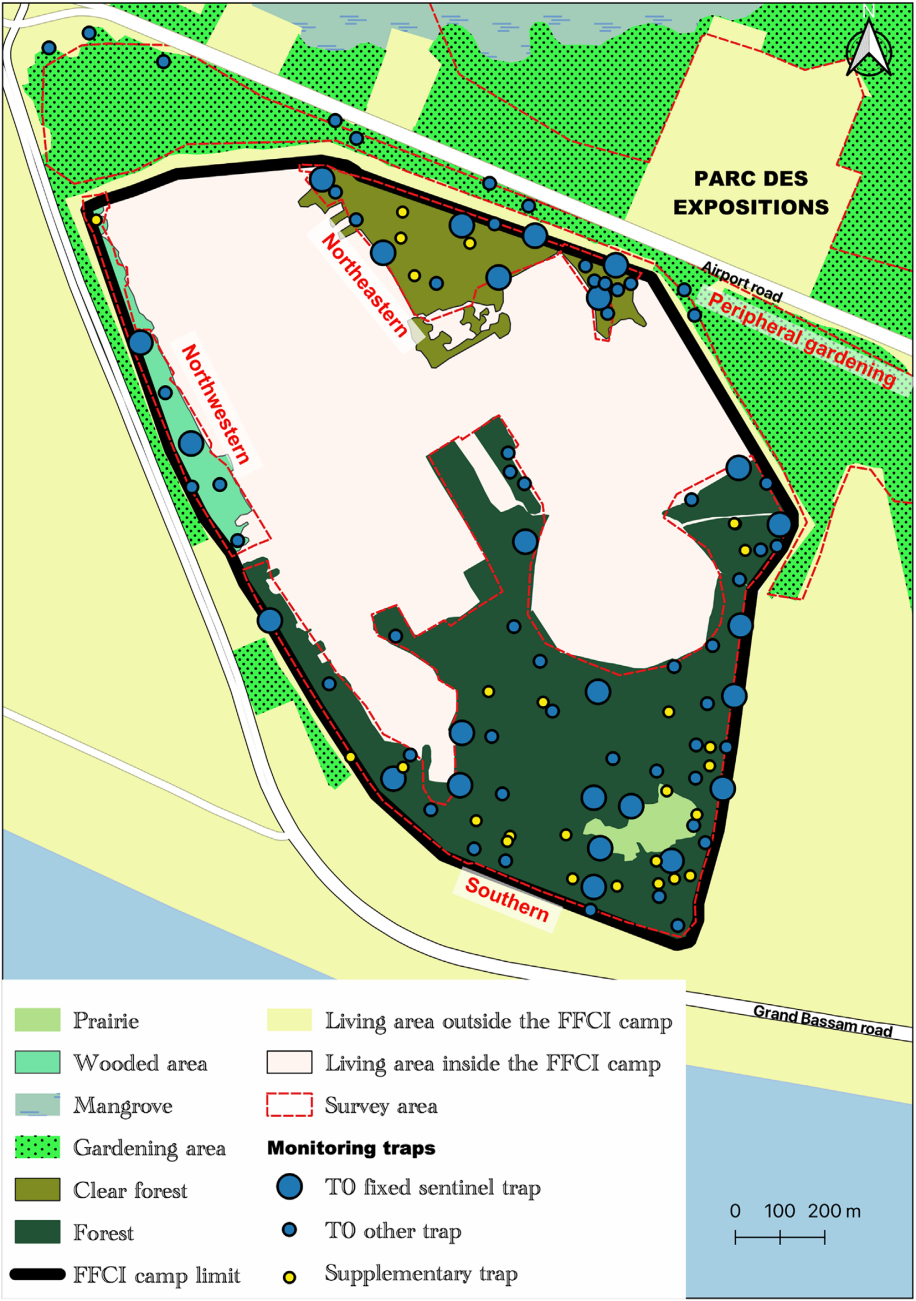
Distribution of vector control monitoring sites (Source: Institut Pierre Richet, 2024).

The main evaluation strategy was based on 25 sentinel sites selected from the T_0_ survey 81 trapping sites. These 25 sites are those where the highest densities of tsetse were observed during the T_0_ survey [46]. They were also selected regarding the distribution of TT from the first deployment and to ensure a homogeneous distribution on the three areas inside the base. A Vavoua trap was set at each of these sites and for all evaluations (T_1_–T_12_) (Fig. 4). These traps were called T_0_ sentinel traps.

One year and two months after the start of VC (T_4_ evaluation, March 2021), we decided to set Vavoua traps at all the 81 sites identified during the initial T_0_ baseline survey to have a more accurate idea of the impact of VC on the distribution of tsetse fly throughout the study area in comparison with T_0_ data. One site (B04) of the T_0_ survey was no longer accessible (restricted area). This evaluation was therefore based on 80 traps including the 25 T_0_ sentinel traps and the 55 other traps called T_0_ other traps (Fig. 4). This evaluation method was repeated one year later (T_8_, March 2022) with traps set at 27 additional sites (supplementary traps) in areas favorable to tsetse fly and not covered by the 80 T_0_ traps (Fig. 4). This operation was then repeated every 3 months (T_9_–T_12_) with the 80 T_0_ traps (25 T_0_ sentinel traps and 55 T_0_ other traps) and the 27 supplementary traps (107 traps in total) until the end of the VC campaign. The number and the status of all traps set for monitoring the VC campaign are detailed in Table 1.

### Infections by trypanosomes

All tsetse flies captured during entomological evaluations were dissected under a binocular stereomicroscope to isolate the proboscis, midgut and salivary glands, which were then examined for the presence of trypanosomes (*Trypanosoma* ssp.) using a compound light microscope at X400 magnification [83].

### Genotyping

Only female tsetse flies were genotyped, as several of the markers used were linked to the X chromosome. Unfortunately, DNA from tsetse flies from the T_0_ survey in January 2020 could no longer be used due to a storage problem. We selected 59 tsetse flies from T_−1_ (May 2019) and 33 tsetse flies captured during the evaluations (from T_1_ to T_12_) whose DNA was available and correctly preserved. The field sampling method consisted of taking three legs from each tsetse fly and storing them in a 1.5 mL tube containing 1 mL of 70% ethanol. In the laboratory, the legs were dried and then treated with Chelex 5% to obtain DNA, as described previously [64].

DNA from each sample was genotyped with eight microsatellite markers: XpGp13 and pGp24 [51], GPCAG [1], B3, XB104, XB110, C102 [42] and pGp20 [65]. The three markers whose names begin with the letter X are located on the X chromosome. Polymerase chain reaction was performed in a thermal cycler in a final volume of 20 μL, using 10 μL of the diluted supernatant from the extraction step as a template. Allele bands were analyzed on an ABI 3500 XL sequencer (Applied Biosystems, Waltham, MA, USA). Allele profiles were read using GeneMapper V 4.1 software (Applied Biosystems) with the GS600LIZ short-term size standard.

### Data analysis

The apparent density per trap per day (ADT), which represents the average number of tsetse flies caught per Vavoua trap per day, was calculated. The rate of trypanosome infection in dissected tsetse flies was also calculated (number of infected tsetse/number of dissected tsetse). The impact of VC through ADT monitoring was studied using a Kruskal–Wallis test. Dunn’s multiple comparison test was used to compare ADTs between evaluation periods. The Fisher exact test was used to study the variation in trypanosome infection rates between the different evaluation periods.

For all population genetic analyses, Create software [14] was used to recode the data into the appropriate format. The GPCAG locus, which had previously been suspected of producing a selection signature in response to VC in another area [5], was analyzed separately from the other seven markers.

We considered a duration of 2 months for a generation (cohort) of tsetse flies, *i.e.*, six generations per year [80]. The base cohort (C0) corresponded to tsetse fly captured on T_−1_ of May 2019. A cohort number (CX) corresponding to the number of generations between T_−1_ and the evaluation period was assigned to each tsetse fly. As such, the tsetse flies caught during the first T_1_ evaluation in June 2020, *i.e.*, 12 months and six generations later, were from cohort C6. The last cohort was that of the tsetse flies captured in T_12_ (March 2023), *i.e.*, 46 months after T_−1_ (C23).

#### Determining the relevant subpopulation unit

We tested three possible hierarchical levels: trap, zone and cohort. We calculated Wright’s identity index *F*_IS_ within individuals relative to the identity between individuals within the same subpopulation [82], which is a measure of the deviation of genotype frequencies from expectations in the case of local panmixia. This parameter was estimated using Weir and Cockerham’s unbiased estimator *f* [79], calculated with Fstat 2.9.4 [34] (updated from Goudet [32]). Using the strategy developed by Goudet [31], we compared this statistic indicator by considering each of these three levels (trap, zone and cohort) as the subpopulation unit. In the case of a significant subdivision at a lower level (for example between traps), we expected an increase in the *F*_IS_ resulting from a Wahlund effect when we considered the next higher level (the zone). We therefore calculated *F*_IS__traps, *F*_IS__zone and *F*_IS__cohort and compared them between each pair using one-tailed Wilcoxon tests for paired data, the unit of matching being the locus. In the case of subdivision (Wahlund effect), we expected (alternative hypothesis) that *F*_IS__traps < *F*_IS__zone < *F*_IS__cohort. These tests were carried out using the R-commander (Rcmdr) package [28, 29] for R version 4.3.1 (2023). For these calculations, we considered sub-samples from different cohorts as different units. When necessary, we used the correction of Benjamini and Hochberg (BH) [3], in order to adjust the *p*-values for the repetition of independent tests, or that of Benjamini and Yekutieli (BY) [4] when the tests were not independent in the series. These adjustments were made using R (p.adjust command).

#### Quality test for loci

We first tested for linkage disequilibrium (LD) between all pairs of loci using the *G*-based test, the most powerful procedure for combining analyses across all subsamples [18]. With *L* loci, we have *k =* (1/2)*L*/(*L* − 1) auto-correlated tests. To correct the false discovery rate (FDR) associated with this series of *k* tests, we used the BY procedure.

We also calculated Wright’s *F*_ST_ and *F*_IT_ [82]. The first is a measure of the identity between individuals in relation to the identity between sub-samples. It therefore measures the effect of subdivision on inbreeding. The second is the inbreeding of individuals relative to the inbreeding of the total population and results from the action of Wright’s two other statistics, *F*_IS_ and *F*_ST_: *F*_IT_ = 1 − (1 − *F*_IS_)(1 − *F*_ST_) [82]. These statistics were estimated using Weir and Cockerham’s unbiased estimators *θ* and *F* [79] with Fstat. Significance of deviation from expectations under local panmixia (*F*_IS_) and subdivision (*F*_ST_) was assessed with 10,000 randomizations of alleles between individuals within subsamples or individuals between sub-samples, respectively. In each case, the statistic used was *f* [79] or *G* [33], respectively. We calculated 95% confidence intervals (95% CI) with 5,000 bootstraps on loci, or jackknives on sub-samples [17]. We also calculated standard errors for *F*_IS_ and *F*_ST_ (SE(*F*_IS_) and SE(*F*_ST_)) using jackknives over the loci. These estimates and randomizations were performed with Fstat 2.9.4. We also calculated the 95% CI of *F*_IS_ with 5,000 bootstraps over individuals for each locus and in each sub-sample using Genetix 4.05.2 [2]. For this last computation, we excluded sub-samples with fewer than five genotyped individuals to avoid execution errors, and averaged the 95% CIs over the sub-samples for each locus.

The effects of null alleles, stuttering and short allele dominance (SAD) were diagnosed using a series of criteria detailed in several articles [16, 19, 21, 22, 53]. The ratio *r*_SE_ = SE(*F*_IS_)/SE(*F*_ST_) > 2, a positive correlation between *F*_IS_ and *F*_ST_ and between the number of missing genotypes (*N*_0_) and *F*_IS_, is a symptom of the presence of null alleles. Correlations were measured and tested using Spearman’s rank correlation test with Rcmdr. We then ran the *F*_IS_ ~*N*_0_ regression and extracted the coefficient of determination *R*^2^ as the proportion of *F*_IS_ variance explained by missing data (putative null homozygotes) and the *F*_IS_ _0 intercept as the putative *F*_IS_ for loci with no missing data (possibly no null alleles). We also used FreeNA [11] to calculate the frequencies of null alleles at each locus in each subsample (*p*_null_*i*_) with the EM algorithm [24]. Missing data were recoded as homozygous for the 999 alleles, as recommended [11], except for loci with too many missing data points, which probably did not correspond to null homozygotes. Expected null homozygotes were then calculated as *N*_*i*_*p*_null_*i*_^2^, where *N*_*i*_ is the size of sub-sample *i*. We then summed these quantities over subsamples for each locus and compared them to the total number of missing data observed for each locus (*N*_0_) using a one-tailed binomial exact test with R (binom.test command, with alternative hypothesis H1 = “there are fewer missing data than expected”). We also calculated the weighted averages of null allele frequencies over the subsamples (*p*_null_ = Σ_*i* _*N*_*i*_*p*_null_*i*_/Σ_*i*_ *N*_*i*_) for each locus and computed the *R*^2^ and *F*_IS_0_ intercept of the corresponding *F*_IS_~*p*_null_ regression.

Stuttering detection between alleles close in size (one or two base pairs) followed the procedure described by De Meeûs and Noûs [22] using the associated Excel template file. We detected SAD with the *F*_IT_ correlation and allele size, using the one-sided Spearman rank correlation test (H1 = negative correlation) [53] with Rcmdr. In the case of significant or marginal values, and to exclude the effect of rare alleles, we then undertook the regression *F*_IS_ ~ Allele size weighted by *p*_*a*_ (1 − *p*_*a*_), where *p*_*a*_ is the frequency of the allele of size *a* [16], with Rcmdr. In the case of a negative slope, the associated *p*-value was then halved to obtain a one-sided probability. At the end of these successive measurements and tests, we excluded outlier loci and corrected for null alleles (with FreeNA) and for stuttering where possible and/or relevant.

#### Genetic differentiation at the GPCAG locus and other loci

To obtain a sufficiently reliable estimate of allele frequencies, we decided to work on sub-samples of size *N* ≥ 5. We therefore used two sub-samples for this test: C0 (T_−1_) (*N* = 59) and C23 (T_12_) (*N* = 18). Due to the presence of null alleles, we used the ENA correction [11] with FreeNA to obtain a corrected estimate of the *F*_ST_ENA_ genetic subdivision with 95%CI using 5000 bootstraps over loci. Given that microsatellites generally exhibit excess polymorphism, we calculated the maximum possible *F*_ST_ of the data after recoding by RecodeData [54], *F*_ST_max_, and used this quantity to obtain standardized estimates of the *F*_ST_ENA_’ *= F*_ST_ENA_/*F*_ST_max_ with its 95% CI. In order to be able to compare values between sub-samples before and after the control, we performed these calculations between pairs of sub-samples. We compared the *F*_ST-ENA_’ at the GPCAG locus with that of the seven other loci described as “neutral” loci (XB110, XB104, C102, pGp20, pGp24, XpGp13, and B3).

#### Global genetic structure before and after VC: correspondence factorial analysis

Given the fragmentary structure of our data after the start of VC, we used an indirect method to assess the effect of VC on the genetic structure of *G. p. palpalis* female population. We performed a factorial correspondence analysis (FCA) [70] using Genetix 4.05 software [2]. In this analysis, each individual is represented by the coordinates of its projection on the plane that minimizes the distances to all the points (individuals). Each axis of each major plane is given a score (inertia) that measures its degree of fit to the data. These inertias correspond to the eigenvalues of the matrix defined by the table as a whole, and the sum of all these eigenvalues gives the trace of the analysis. The percentage of inertia can then be calculated for each axis in relation to this trace. According to the Genetix web page (https://kimura.univ-montp2.fr/genetix/), with a matrix of allele occurrences (0, 1, or 2) for each allele at each locus and each individual, the trace of the corresponding matrix is linked to Robertson and Hill’s *F*_ST_ [68]. Consequently, the values along each principal axis can be assimilated to linear combinations of allelic *F*_STs_ [36]. We assessed the significance of each axis using the broken-stick method [30].

Genetic diversity was measured in subsamples from the French military base collected before (C0) and after control (CX; we pooled all subsamples collected after control) with Nei’s *H*_S_. Pairwise comparisons were performed with the Wilcoxon test for paired data with Rcmdr, the matching units being loci, and with an alternative hypothesis that *H*_S_ should be higher before than after the start of vector control. For this comparison, we used the seven neutral loci.

#### Estimation of tsetse fly effective population sizes

The effective population size was estimated by the heterozygote excess method [23] (HE), the LD method [77] taking into account the effects of missing data [62], the coancestry method [60] (CA), the intra- and inter-loci identity probabilities [74] (1&2L), and the sibship frequencies (SF) method [76]. For HE, we simply used Weir and Cockerham’s *F*_IS_ estimate in the formula $N_{e}=-\frac{1}{2F_{\mathrm{IS}}}-\frac{F_{\mathrm{IS}}}{2\left(1+F_{\mathrm{IS}}\right)}$ for each locus and in each sub-sample, and then calculated the mean of the values at the loci for each sub-sample [23]. For LD and CA, we used NeEstimator [27]. For 1&2L, we used Estim [75]. Finally, for SF, we used Colony [38]. For LD, we chose the “*random mating*” option and used estimates ignoring all alleles with frequencies below 0.05 as recommended in the NeEstimator manual. As described elsewhere [23], we calculated the average, weighted by the number of useful values (other than “infinity”) over all methods.

#### Bottleneck signatures

Finally, we also looked for the genetic signature of a past bottleneck [15] using Bottleneck software [63]. As recommended in De Meeûs’s book on page 109 [20], we used the three mutation models: IAM, TPM (with default options), and SMM. Significant signatures of a bottleneck can be suspected if the test is highly significant with IAM and significant with TPM at least. Weakly significant tests tend to be produced in populations with small effective sizes (De Meeûs, unpublished simulation results). Significance was assessed using the Wilcoxon rank test, as recommended [15].

## Results

This study specifically describes the results obtained during the VC campaign implemented at the French military base, comparing them with those of the entomological baseline surveys carried out in May 2019 (T_−1_) and January 2020 (T_0_) on the ecology of tsetse flies in this zone [46].

### Entomological surveys carried out in the area around the French military base

No tsetse flies were caught by the entomological surveys carried out in June and September 2020 in the peripheral gardening area, the Ebrié lagoon gardening and mangrove area, and the Adjahui-Coubé zone. Furthermore, no tsetse flies were caught by the T_0_ other traps set in the peripheral gardening area during the six evaluations carried out from March 2021 (T_4_) to March 2023 (T_12_).

### Monitoring of VC

The raw data on the number of tsetse flies caught during the 12 entomological evaluations, including the 25 T_0_ sentinel traps, the 55 T_0_ other traps and 27 supplementary traps, are described in Supplementary material 1. Regarding the 25 T_0_ sentinel traps, the Kruskal–Wallis test showed a significant difference in ADT between the evaluation periods (*p* = 2.2e-16) over the entire study area. It fell from 5.47 tsetse fly/trap/day at T_0_ to 0.08 at T_1_, showing a significant reduction of 98.53% (*p* < 0.0001). ADT obtained during the subsequent evaluations T_2_ to T_12_ remained low and ranged from 0 to 0.13 tsetse fly/trap/day, all significantly lower than the T_0_ ADT (*p* < 0.0001).

**Figure 5:**
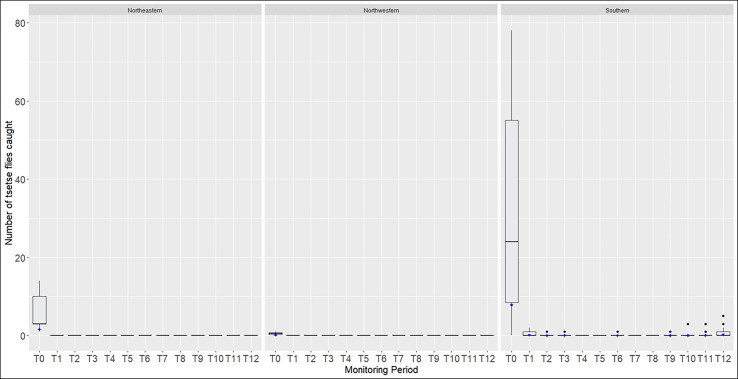
Boxplot of the number of captured tsetse flies during the entomological evaluation in the 25 T_0_ sentinel traps according to zones. The boxplot (vertical bars in grey) shows the median and interquartile range, the whiskers show the 10th and 90th centiles and the black dots show all values >90th centile. The average catches per trap and per day (ADT) are represented by the blue dots.

Taking zone into account, no tsetse flies were captured by the T_0_ sentinel traps in the northwest and northeast zones from T_1_ to T_12_ (Fig. 5). With the 16 T_0_ sentinel traps set in the southern zone, we confirmed the significant decrease in ADT between T_0_ (7.84 tsetse fly/trap/day) and T_1_ (0.125 tsetse fly/trap/day) (*p* < 0.0001). In this zone where most of the tsetse flies were captured, ADT from T_2_ to T_12_ remained low and lower than the at T_0_ (*p* < 0.0001). However, after T_8_ (no tsetse fly captured), a gradual re-increase in ADT was observed, which became significantly higher at T_12_ (0.203 tsetse/trap/day; *p* = 0.007).

**Figure 6:**
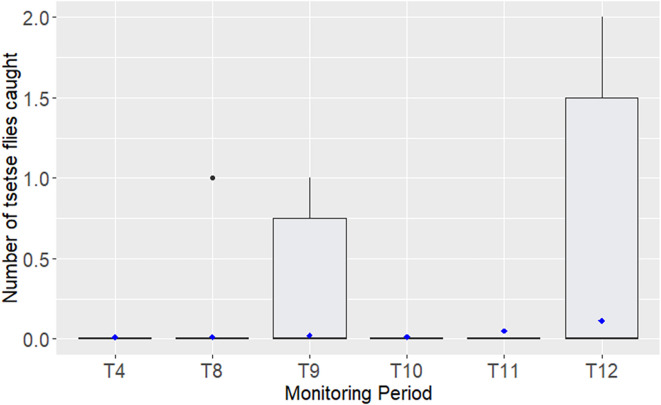
Boxplot of the number of captured tsetse flies with the T_0_ and supplementary traps. The boxplot (vertical bars in grey) shows the median and interquartile range, the whiskers show the 10th and 90th centiles and the black dots show all values >90th centile. The average catches per trap and per day (ADT) are represented by the blue dots.

Results obtained with the 80 T_0_ traps (25 T_0_ sentinel traps and 55 T_0_ other traps) over the whole study area confirmed the significant drop in ADT between T_0_ (2.38 tsetse fly/trap/day) and T_4_ (0.0093 tsetse fly/trap/day) (*p* < 0.0001). Monitoring carried out with these 80 T_0_ traps and the 27 supplementary traps added at T_8_ also showed low ADTs of less than 0.028 tsetse fly/trap/day from T_8_ to T_11_. It also confirmed the increase in ADT at T_12_, reaching 0.081 tsetse flies per trap per day (*p* < 0.0001) (Fig. 6). It should be noted that the T_0_ other traps and supplementary traps captured tsetse flies at T_4_ and T_8_ evaluations, while the T_0_ sentinel traps did not capture any. The only tsetse fly observed at T_12_ in the northeastern zone was captured by a T_0_ other trap (Supplementary material 1).

### Infections by trypanosomes

The raw data on trypanosome infections (without distinction of species) in captured and dissected tsetse flies are shown in Supplementary material 1. All infections observed during the VC campaign were detected in the southern area. The number of trypanosome infected tsetse flies and infection rates per evaluation are given in Table 2. Only five tsetse flies were found to be infected during the vector campaign (one at T_1_, T_6_, T_9_, T_11_ and T_12_). However, the number of dissected flies was low (ranging from 0 to 12). The Fisher exact test showed no significant difference (*p* = 0.83) in infection rates between the different entomological evaluation periods. It is important to note that there were still two infected flies at the end of the campaign (T_11_ and T_12_).

**Table 2:** Number of trypanosome-infected tsetse flies per evaluation.

Monitoring	Dissected	Infection	Infection rate (%)
T_0_	42	10	23.8
T_1_	8	1	12.5
T_2_	2	0	0
T_3_	3	0	0
T_4_	0	0	0
T_5_	0	0	0
T_6_	2	1	50
T_7_	0	0	0
T_8_	0	0	0
T_9_	2	1	50
T_10_	3	0	0
T_11_	4	1	25
T_12_	12	1	8.33

### Population genetics

The raw data obtained with the eight microsatellite markers on the sample of 92 tsetse flies selected for genotyping are described in Supplementary material 2. Our sample contained the following tsetse cohorts: C0 (T_−1_, *n =* 59), C6 (T_1_, *n =* 4), C8 (T_2_, *n =* 1), C14 (T_6_, *n =* 1), C18 (T_9_, *n =* 2), C20 (T_10_, *n =* 2), C21 (T_11_, *n =* 5) and C23 (T_12_, *n =* 18).

#### Subdivision levels

No Wahlund effect could be demonstrated with the seven neutral loci by grouping traps within zones or zones within cohorts (smallest *p* = 0.1167) (Supplementary material 3). For the analyses to follow, we therefore considered the cohorts as sub-sample units.

#### Quality of loci

No pairs of loci were in significant LD with the seven neutral loci (minimum *p* = 0.14). Overall, we observed a significant heterozygote deficit that varied from one locus to another (Supplementary material 4).

There were indications of the presence of null alleles with *r*_SE_ = 17.6, a correlation between *F*_IS_ and *F*_ST_ (*ρ* = 0.4685; *p* = 0.1445), and a correlation between *F*_IS_ and the number of missing data *N*_0_ (*ρ* = 0.1261; *p* = 0.3938), which was positive but not significant. Some loci had too many missing genotypes compared with what was expected, based on the *F*_IS_ observed at these loci (XB110, XB104 and pGp24 loci, Supplementary material 5). We therefore recoded all the missing data into homozygotes for null alleles (coded 999), except for these three loci, and used FreeNA to estimate the frequencies of null alleles for each locus. The *F*_IS_ ~ *p*_null_ regression resulting from these analyses showed a good fit (*R*^2^ = 0.96, *F*_IS_0_ = 0.0023) (Supplementary material 6).

With regard to stuttering, only locus B3 showed a significant *p*-value. However, it was not possible to correct for stuttering at this locus without grouping all the alleles. Nevertheless, this locus was very well explained by the null alleles. None of the loci showed a significant SAD.

#### Genetic differentiation at the GPCAG locus and other loci

**Figure 7:**
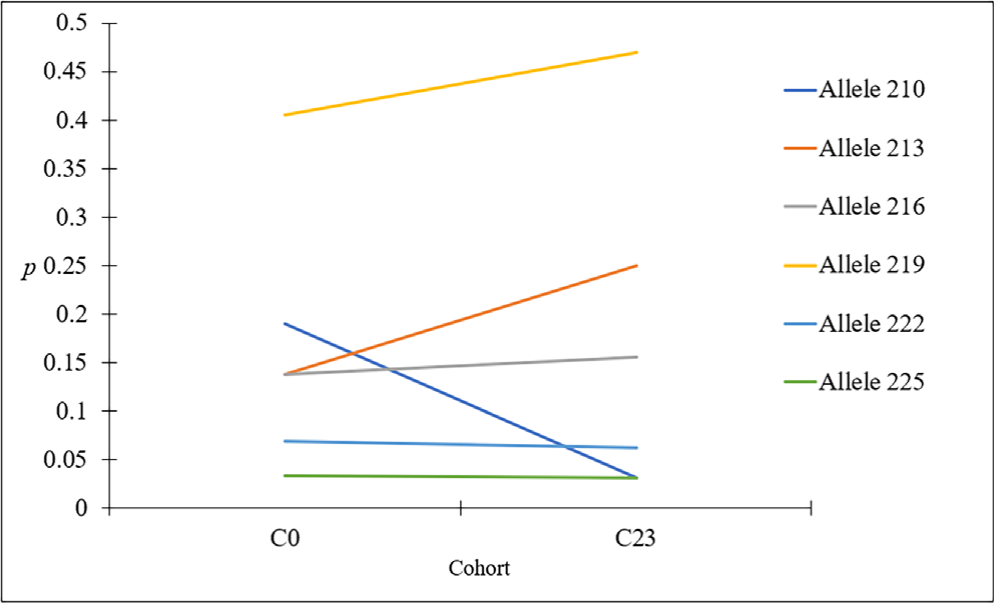
Frequencies of the main alleles (219, 213, 210, 216, 222, and 225) at the GPCAG locus before and after the VC campaign. C0: Baseline entomological survey in May 2019; C23: Entomological evaluation in March 2023 (more than 3 years after the implementation of the VC).

The GPCAG locus (*F*_ST_ENA_GPCAG_’ = 0.0413; *p* = 0.17) showed higher subdivision than the mean and 95%CI of the seven neutral loci (*F*_ST_ENA_neutral_loci_’ *=* −0.00095; 95% CI *=* [−0.0435, 0.0407]; *p* = 0.16). With these seven neutral loci, no temporal subdivision was observed. Changes in allele frequencies revealed that this subdivision was mainly due to a 15.80% increase in the 219 allele (from 0.405 to 0.469), a 60% increase in the 213 allele (0.138 to 0.25) and an 84% decrease in the 210 allele (0.19 to 0.031) at the GPCAG locus after treatment (Fig. 7). It should be noted that allele 219, which appeared under strong positive selection in the focus of Bonon [5] already had a high allele frequency (*p*_219_ = 0.405) before the start of the VC in January 2020. The GPCAG locus, potentially under selection [5], was then excluded from the following analyses.

#### Global genetic structure before and after the beginning of control measures

**Figure 8:**
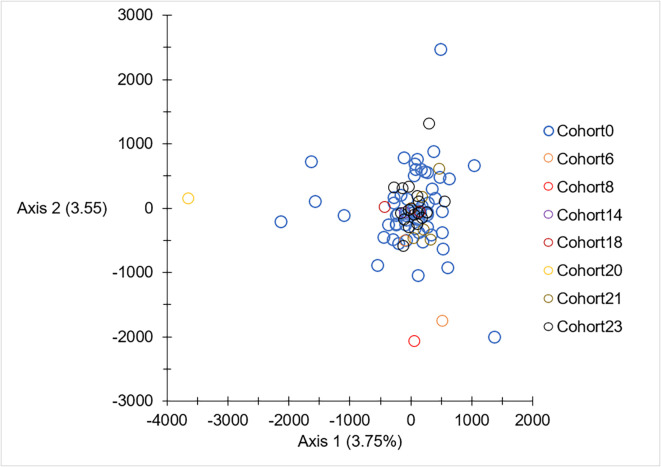
Factorial Correspondence Analysis obtained with the seven neutral loci. CohortX: Individuals are represented by empty circles of different colors (one color for one cohort).

The results of the factorial correspondence analysis (FCA), without GPCAG, are shown in Figure 8. No axis was significant, confirming the absence of significant temporal subdivision, as described above. All the individuals caught after the start of VC are contained in the cloud defined by those caught before the control, except for one C20 individual. The fact that one individual from C20 deviated from the main cloud was due to an excess of missing data found (4 of 7 loci).

Genetic diversity increased or decreased slightly depending on the locus after the implementation of VC, but no significant difference was found between the sub-samples captured before (C0) and after VC (*p* = 0.223). The effective population size decreased after the start of VC for the methods that gave usable values for both cohorts: coancestries from *N*_*e*_ *= x* to *N*_*e*_ *= y*; and sibship from *N*_*e*_ *= z* to *N*_*e*_ *= t*, which is probably not significant, or weakly so (Table 3).

**Table 3:** Comparisons of effective population sizes for the C0 and C23 cohorts.

Cohort	HzES	LD	Coancestries	Sibship	Average
C0	48.8201	1180.3	42.9	73	336.255
C23	Infinite	Infinite	27.3	25	26.15
Average	48.8201	1180.3	35.1	49	181.2025

HzES: Excess heterozygote; LD: linkage disequilibrium.

#### Bottleneck signatures

The results of the bottleneck analyses are presented in Table 4. A weakly significant signal was observed only with the IAM model. We cannot, therefore, reject the null hypothesis and conclude that there was no bottleneck signature, particularly after 19 generations of intensive control measures between the first deployment and the last T_12_ evaluation in March 2023.

**Table 4:** Results of tests for the detection of a genetic signature of a bottleneck with the three mutation models (IAM, TPM and SMM) for sub-samples before the start (C0) and after vector control (C23).

Cohort	IAM	TPM	SMM
C0	0.0390	0.1875	0.9609
C23	0.0273	0.1484	0.5937

## Discussion

The aim of the VC campaign carried out against *G. p. palpalis* at the French military base was to reduce the risk of trypanosome transmission, or even to interrupt it permanently by using TTs. This appeared to be the only control strategy available and acceptable in this urban and preserved environment. To achieve this, it was important to assess the risk of tsetse fly immigration from a neighboring area. Entomological surveys carried out in biotopes identified as potentially favorable to these vectors on the outskirts and in the vicinity of the study area, during which no tsetse were captured, support the view that the tsetse population at the base is geographically isolated. The results of the population genetics study confirmed strictly local recruitment of flies after the start of the control program, and therefore an absence of immigrant flies from outside the camp.

The various methods used to assess the impact of VC on the tsetse fly population at the base confirmed that TTs can rapidly and effectively reduce tsetse density (over 98% reduction in just a few months). This effectiveness had already been demonstrated for the same species, *G. p. palpalis*, in the Bonon HAT outbreak in Côte d’Ivoire [39] as well as for other tsetse species in various HAT foci in West and Central Africa [42, 52, 55, 71, 72]. Although these VC campaigns combined with medical control have proved effective, particularly in Bonon in Côte d’Ivoire, there are still questions about the sustainability of the results after VC is discontinued [40].

At the base, for obvious ecological reasons, it was not feasible to control the trypanosome reservoir, which consists mainly of protected wild animal species [46]. However, it seemed realistic to us that the risk of transmission could be interrupted in the long term by using only VC, with multiple TT replacements and reinforcements, given the probable isolation of the target population. By using a much higher density of TT/km^2^ than usual when compared to past studies, we were able to maintain a very low tsetse density throughout the control period. This was achieved even in the southern zone, where the most favorable biotopes are located, and despite control being forbidden in parts of it classified as protected areas. However, we observed a gradual then significant re-increase in ADT during the last evaluation in March 2023 (T_12_) only 11 months after the last massive redeployment of TTs. During this last evaluation, a tsetse fly was even captured in the northern part of the base (northeastern zone) where no captures had been observed since the introduction of VC. While VC has had a rapid and significant impact on tsetse fly densities, it has not eliminated them despite the efforts made. As a result, the end of VC was rapidly accompanied by the return of tsetse flies and recolonization of the area. Unfortunately, this cessation also led to a resumption of trypanosome transmission, which could not be interrupted during the campaign. We should note that since the species of trypanosomes observed in tsetse flies were not identified, we can only assess the impact of our intervention on the overall infection rates, which is a limitation of our study.

There are several possible explanations for the difficulties encountered in eliminating an apparently isolated tsetse fly population in such a small geographical area. One of them is the fact that the military base has an area of preserved and protected forest in its southern part, which in places is very difficult to access. The desire to preserve this environment made it difficult, if not impossible, to install TTs in protected areas. Additionally, in places where TTs were installed directly within the vegetation, their effectiveness was limited due to reduced visibility, which decreased their attractiveness to tsetse flies. These areas are particularly favorable biotopes for tsetse flies, with the constant presence of tsetse hosts such as the bushbuck (*Tragelaphus scriptus*), which has been reported as the main host of *G. p. palpalis* [10, 13]. In this type of biotope, tsetse flies do not need to fly very much, whether to feed or to reproduce, reducing the probability of coming into contact with a TT. In fact, most of the captures observed during the evaluations were made in the vicinity of such sites. It is probably in these areas that trypanosome transmission from a wild animal reservoir is maintained in a highly localized fashion. It is therefore clear that these biotopes, combining favorable conditions of vegetation and humidity, and the availability of hosts, explain the ability of this tsetse population to survive at apparent low densities. This also explains the limited impact of control with TTs on the elimination of this tsetse fly population and even on the possibility of interrupting trypanosome transmission.

Population genetic analyses revealed another constraint that may explain the difficulty in eliminating the tsetse fly population from the military base. The fact that genetic diversity remained unchanged after VC, that effective population sizes were weakly affected (if any) and the absence of any bottleneck signature even after 19 generations, support the existence of a large and stable tsetse population, despite control efforts. Moreover, highly significant differentiation was observed between sub-samples before and after the start of VC for the GPCAG locus. A study carried out in the Bonon HAT outbreak in Côte d’Ivoire has already shown that this locus was possibly correlated with VC measures, with a significant increase in frequency of allele 219 seven generations after the start of control, followed by stabilization between 0.48 and 0.56 [5]. Our study confirms the non-neutrality of this locus, with an already high frequency of 0.4 of this allele before the start of VC. This high value could be due, among other possibilities, to a previous VC campaign carried out at the base in 2004 and 2005, which had to be interrupted because of the socio-political crisis that occurred in Côte d’Ivoire at the time. During this campaign, a 99% reduction in ADT was observed after six months of control using 26 Vavoua traps and 68 conventional screens impregnated with deltamethrin (Kaba *et al*., unpublished data). The selection of particular alleles, such as allele 219 at the GPCAG locus, suggests that tsetse fly populations might adapt to VC by one or more mechanisms that remain to be investigated (resistance to deltamethrin, avoidance of traps or other), as already reported [5]. The continued high frequency of this allele in the absence of VC between 2005 and 2020 suggests that there is no cost to this allele in the absence of VC, as is the case for insecticide resistance in insects [12].

In this study, we showed that TTs alone, even if deployed at very high densities, were not able to eliminate a tsetse fly population isolated in an area of less than 2 km^2^, despite all the control and evaluation efforts deployed over three years of VC. This result means that the addition of natural and TT-based mortality rates probably remained smaller than the birth rate of tsetse. This is partly due to a biotope that is particularly favorable to tsetse flies in terms of habitat and host availability, explaining a large part of the resilience of this population, but may also be due to the adaptability of this species, as suggested by the results on the GPCAG locus. Further work will be needed to study the mechanisms of this adaptation, including behavioral studies. Nevertheless, the TTs that are particularly well adapted to the conserved environment of the base considerably reduced the risk of trypanosome transmission to animals and humans. We suggest that this strategy be pursued in order to continue to limit the risk of transmission, and that further thought be given to the strategies to be implemented in order to eliminate the tsetse fly population in this area on a long-term basis, in particular by combining other control tools such as FlyScreens, which have recently been developed to control trypanosome vectors [25]. Eliminating a tsetse population (as small and isolated as it may be) using toxic and immobile attractants remains a challenge in the history of tsetse fly control. Other biological control strategies could be considered, such as satyrization [56] or the sterile insect technique to achieve local eradication. The sterile insect technique that recently helped to eliminate a large population of *G. p. gambiensis* in the Niayes region of Senegal [69] could be applied, while taking care to protect human populations from the nuisance of tsetse bites. Furthermore, this would involve many challenges for such a reduced geographical scale, including the technical challenge of producing sterile males of *G. p. palpalis.*

## Supplementary material

The supplementary material for this article can be found at https://www.parasite-journal.org/10.1051/parasite/2025017/olm. *Supplementary material 1*: Entomological database. Traps Nb: Number of each trap PGE: Peripheral Gardening Area TotalGloss_TxMonthYear: Total number of tsetse flies captured per period of entomological monitoring (Tx) per month and per year ADT_TxMonthYear: Apparent Density per Trap per day per period of entomological monitoring (Tx) per month and per year Infect_T0MonthYear: Number of infected tsetse flies per period of entomological monitoring (Tx) per month and per year NS: Not Set T_0_: Trap set on sites identified during the January 2020 initial entomological survey *Supplementary material 2*: Genotyping data base FlyNb: Number of each tsetse fly Tx: Entomological monitoring XpGp13 to GPCAG: microsatellite loci *Supplementary material 3*: *F*_IS_ results estimated within traps, zones or cohorts *F*_IS_ are shown for each locus. The average *F*_IS_ for all loci is represented by empty black circles. *Supplementary material 4*: Comparison of *F*_IS_ between neutral loci *F*_IS_ values are represented by solid black circles, and their 95% confidence intervals by dashes (bootstrap on individuals for each locus or on loci (All)). Significant deviations from panmictic expectations, the number of observed and expected missing data (in brackets) and the results of the stuttering test are also provided. *Supplementary material 5*: *F*_IS_ regression as a function of the number of missing genotypes Regression of *F*_IS_ against the number of missing genotypes with neutral loci only is represented by the single line with 95% CI bootstraps on individuals (*F*_IS___i_ et *F*_IS___s_, dashed lines). All equations are given and the coefficient of determination (*R*^2^) is provided for the mean. Results for the XB110, XB104 and pGp24 loci are represented by an empty circle, a full circle and an empty square, respectively (black dashes represent 95% CIs). They do not participate in the regression. The other loci are represented by crosses. *Supplementary material 6*: Regression of *F*_IS_ as a function of null allele frequencies (*p*_nulls_) obtained with neutral loci only The 95% CI of bootstraps on individuals (dotted lines) (*F*_IS___i_ and *F*_IS___s_). All equations are given and the coefficient of determination (*R*^2^) is provided for the mean.

## Notes

**Cite this article as**: Konan YJR, Ta BTD, Berté D, Coulibaly B, Coulibaly KD, Egnankon NS, Diarrassouba F, Djabo KA, Watier-Grillot S, Demoncheaux J-P, De Marie Kouadio KA, N’Dri L, Solano P, Ravel S, Adingra GP, Barreaux A, Ségard A, Kaba D, Jamonneau V, De Meeûs T & Djohan V.2025. Challenges and lessons from a vector control campaign targeting *Glossina palpalis palpalis* in an isolated protected forest area in Abidjan, Côte d’Ivoire. Parasite **32**, 25. https://doi.org/10.1051/parasite/2025017.
