# Supplementary figures and images for: Challenges and lessons from a vector control campaign targeting Glossina palpalis palpalis in an isolated protected forest area in Abidjan, Côte d’Ivoire
Source: Parasite. 2025 Apr 15;32:25. doi: 10.1051/parasite/2025017 (PMC12002674; doi:10.1051/parasite/2025017)

## Slide 1
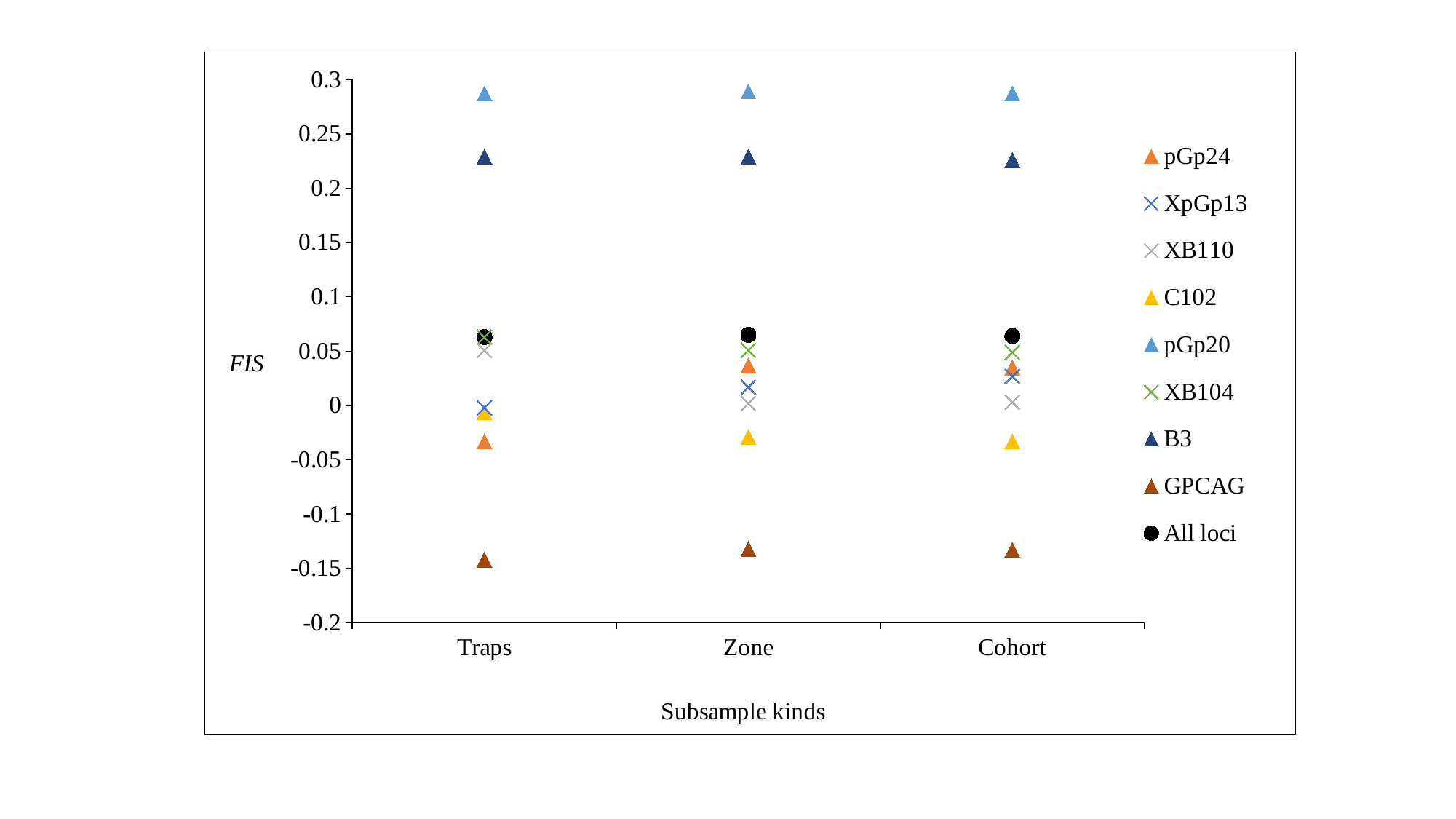

### Chart
| Category | pGp24 | XpGp13 | XB110 | C102 | pGp20 | XB104 | B3 | GPCAG | All loci |
|---|---|---|---|---|---|---|---|---|---|
| Traps | -0.033 | -0.002 | 0.051 | -0.006 | 0.287 | 0.063 | 0.229 | -0.142 | 0.063 |
| Zone | 0.037 | 0.017 | 0.002 | -0.029 | 0.289 | 0.051 | 0.229 | -0.132 | 0.065 |
| Cohort | 0.035 | 0.027 | 0.003 | -0.033 | 0.287 | 0.049 | 0.226 | -0.133 | 0.064 |

Supplement: Supplementary file 1 — Supplementary material 1: Entomological database. Traps Nb: Number of each trap PGE: Peripheral Gardening Area TotalGloss_TxMonthYear: Total number of tsetse flies captured per period of entomological monitoring (Tx) per month and per year ADT_TxMonthYear: Apparent Density per Trap per day per period of entomological monitoring (Tx) per month and per year Infect_T0MonthYear: Number of infected tsetse flies per period of entomological monitoring (Tx) per month and per year NS: Not Set T0: Trap set on sites identified during the January 2020 initial entomological survey Supplementary material 2: Genotyping data base FlyNb: Number of each tsetse fly Tx: Entomological monitoring XpGp13 to GPCAG: microsatellite loci Supplementary material 3: FIS results estimated within traps, zones or cohorts FIS are shown for each locus. The average FIS for all loci is represented by empty black circles. Supplementary material 4: Comparison of FIS between neutral loci FIS values are represented by solid black circles, and their 95% confidence intervals by dashes (bootstrap on individuals for each locus or on loci (All)). Significant deviations from panmictic expectations, the number of observed and expected missing data (in brackets) and the results of the stuttering test are also provided. Supplementary material 5: FIS regression as a function of the number of missing genotypes Regression of FIS against the number of missing genotypes with neutral loci only is represented by the single line with 95% CI bootstraps on individuals (FIS_i et FIS_s, dashed lines). All equations are given and the coefficient of determination (R2) is provided for the mean. Results for the XB110, XB104 and pGp24 loci are represented by an empty circle, a full circle and an empty square, respectively (black dashes represent 95% CIs). They do not participate in the regression. The other loci are represented by crosses. Supplementary material 6: Regression of FIS as a function of null allele frequencies (pnulls) [file parasite-32-25-s1.zip › parasite240104-1-olm/Suppl_mat_3_Fis_trap_zone_cohort.pptx]

## Slide 1
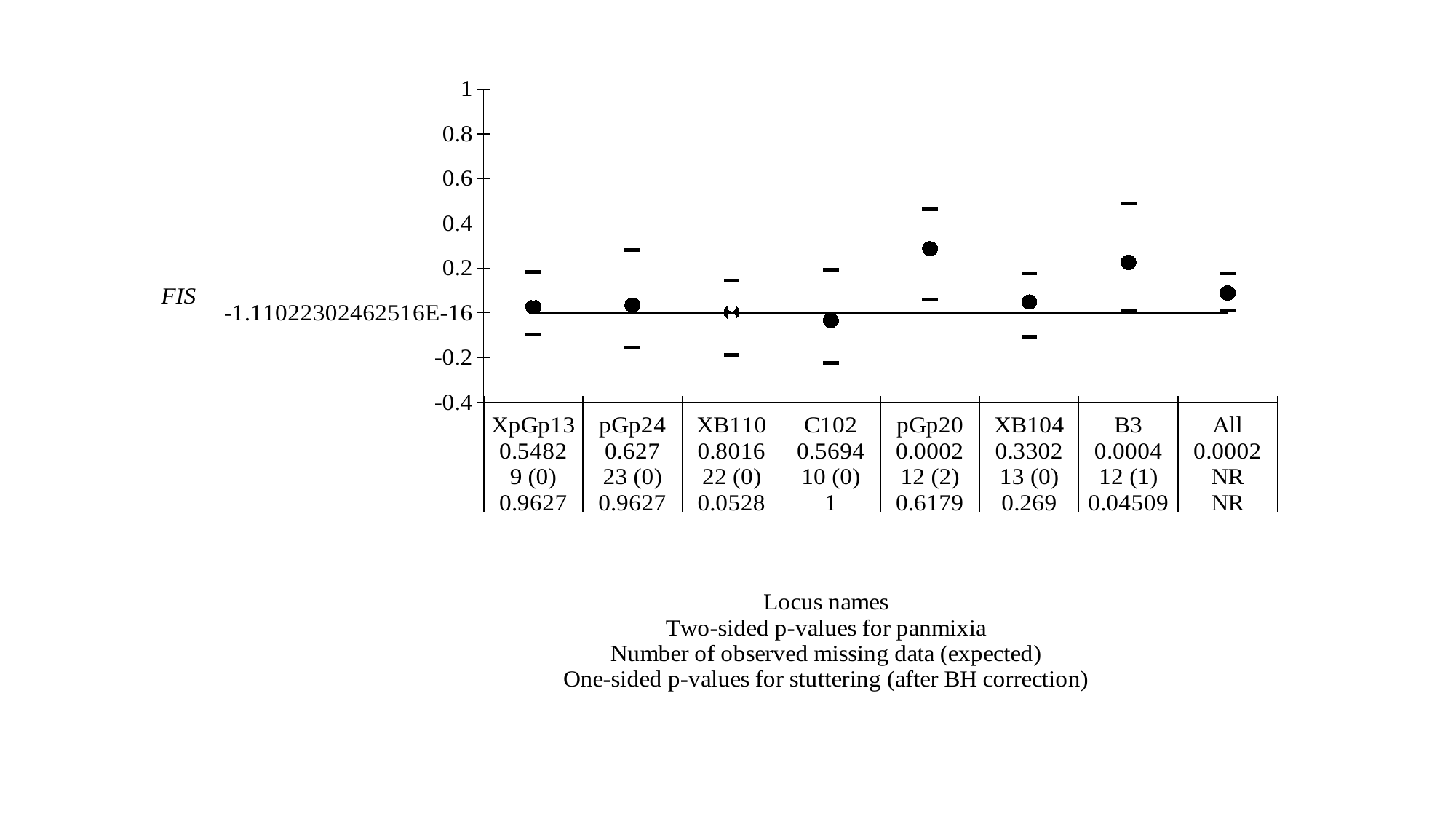

### Chart
| Category | FIS | FIS_i_J | FIS_s_J | FIS_i_B | FIS_s_B | zero |
|---|---|---|---|---|---|---|
| XpGp13 | 0.027 | -0.0194913251717544 | 0.0734913251717544 | -0.097325 | 0.18313 | 0.0 |
| pGp24 | 0.035 | -0.231713391774802 | 0.301713391774802 | -0.15475 | 0.28092 | 0.0 |
| XB110 | 0.003 | -0.0190222066603047 | 0.0250222066603047 | -0.18615 | 0.143165 | 0.0 |
| C102 | -0.033 | -0.0843851488740444 | 0.0183851488740444 | -0.222955 | 0.19202 | 0.0 |
| pGp20 | 0.287 | 0.0912470519084024 | 0.482752948091597 | 0.05991 | 0.462905 | 0.0 |
| XB104 | 0.049 | -0.0978147110686982 | 0.195814711068698 | -0.105615 | 0.17682 | 0.0 |
| B3 | 0.226 | -0.0945454524999911 | 0.546545452499991 | 0.011775 | 0.489185 | 0.0 |
| All | 0.089 | -0.0260048570038136 | 0.204004857003814 | 0.012 | 0.176 | 0.0 |

Supplement: Supplementary file 1 — Supplementary material 1: Entomological database. Traps Nb: Number of each trap PGE: Peripheral Gardening Area TotalGloss_TxMonthYear: Total number of tsetse flies captured per period of entomological monitoring (Tx) per month and per year ADT_TxMonthYear: Apparent Density per Trap per day per period of entomological monitoring (Tx) per month and per year Infect_T0MonthYear: Number of infected tsetse flies per period of entomological monitoring (Tx) per month and per year NS: Not Set T0: Trap set on sites identified during the January 2020 initial entomological survey Supplementary material 2: Genotyping data base FlyNb: Number of each tsetse fly Tx: Entomological monitoring XpGp13 to GPCAG: microsatellite loci Supplementary material 3: FIS results estimated within traps, zones or cohorts FIS are shown for each locus. The average FIS for all loci is represented by empty black circles. Supplementary material 4: Comparison of FIS between neutral loci FIS values are represented by solid black circles, and their 95% confidence intervals by dashes (bootstrap on individuals for each locus or on loci (All)). Significant deviations from panmictic expectations, the number of observed and expected missing data (in brackets) and the results of the stuttering test are also provided. Supplementary material 5: FIS regression as a function of the number of missing genotypes Regression of FIS against the number of missing genotypes with neutral loci only is represented by the single line with 95% CI bootstraps on individuals (FIS_i et FIS_s, dashed lines). All equations are given and the coefficient of determination (R2) is provided for the mean. Results for the XB110, XB104 and pGp24 loci are represented by an empty circle, a full circle and an empty square, respectively (black dashes represent 95% CIs). They do not participate in the regression. The other loci are represented by crosses. Supplementary material 6: Regression of FIS as a function of null allele frequencies (pnulls) [file parasite-32-25-s1.zip › parasite240104-1-olm/Suppl_mat_4_Fis_Locus.pptx]

## Slide 1
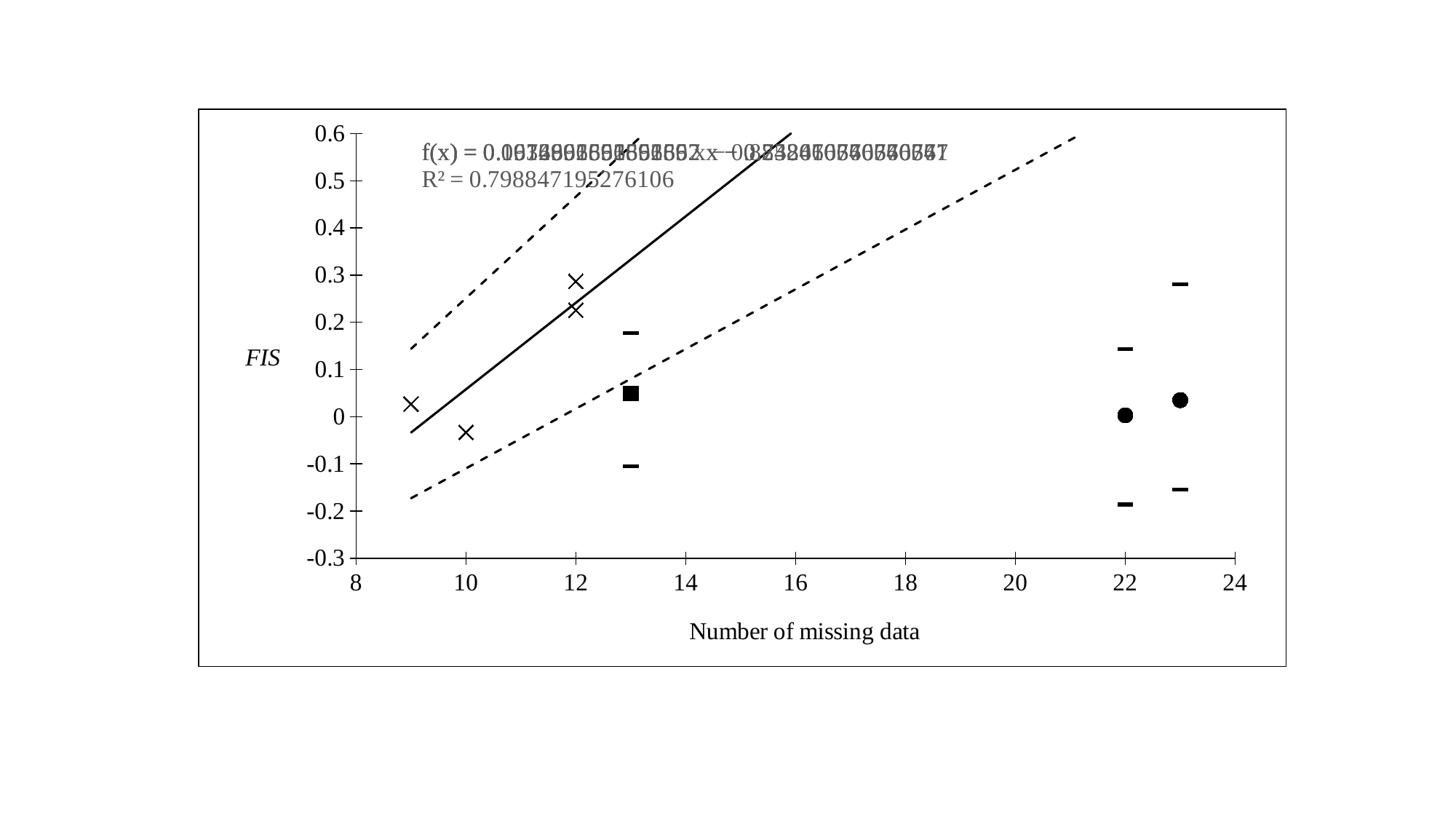

### Chart
| Category | FIS | FIS_i_B | FIS_s_B | Fis_XB110 | li | ls | Fis_pGp24 | li | ls | Fis_XB104 | li | ls |
|---|---|---|---|---|---|---|---|---|---|---|---|---|

Supplement: Supplementary file 1 — Supplementary material 1: Entomological database. Traps Nb: Number of each trap PGE: Peripheral Gardening Area TotalGloss_TxMonthYear: Total number of tsetse flies captured per period of entomological monitoring (Tx) per month and per year ADT_TxMonthYear: Apparent Density per Trap per day per period of entomological monitoring (Tx) per month and per year Infect_T0MonthYear: Number of infected tsetse flies per period of entomological monitoring (Tx) per month and per year NS: Not Set T0: Trap set on sites identified during the January 2020 initial entomological survey Supplementary material 2: Genotyping data base FlyNb: Number of each tsetse fly Tx: Entomological monitoring XpGp13 to GPCAG: microsatellite loci Supplementary material 3: FIS results estimated within traps, zones or cohorts FIS are shown for each locus. The average FIS for all loci is represented by empty black circles. Supplementary material 4: Comparison of FIS between neutral loci FIS values are represented by solid black circles, and their 95% confidence intervals by dashes (bootstrap on individuals for each locus or on loci (All)). Significant deviations from panmictic expectations, the number of observed and expected missing data (in brackets) and the results of the stuttering test are also provided. Supplementary material 5: FIS regression as a function of the number of missing genotypes Regression of FIS against the number of missing genotypes with neutral loci only is represented by the single line with 95% CI bootstraps on individuals (FIS_i et FIS_s, dashed lines). All equations are given and the coefficient of determination (R2) is provided for the mean. Results for the XB110, XB104 and pGp24 loci are represented by an empty circle, a full circle and an empty square, respectively (black dashes represent 95% CIs). They do not participate in the regression. The other loci are represented by crosses. Supplementary material 6: Regression of FIS as a function of null allele frequencies (pnulls) [file parasite-32-25-s1.zip › parasite240104-1-olm/Suppl_mat_5_Fis_missing_data.pptx]

## Slide 1
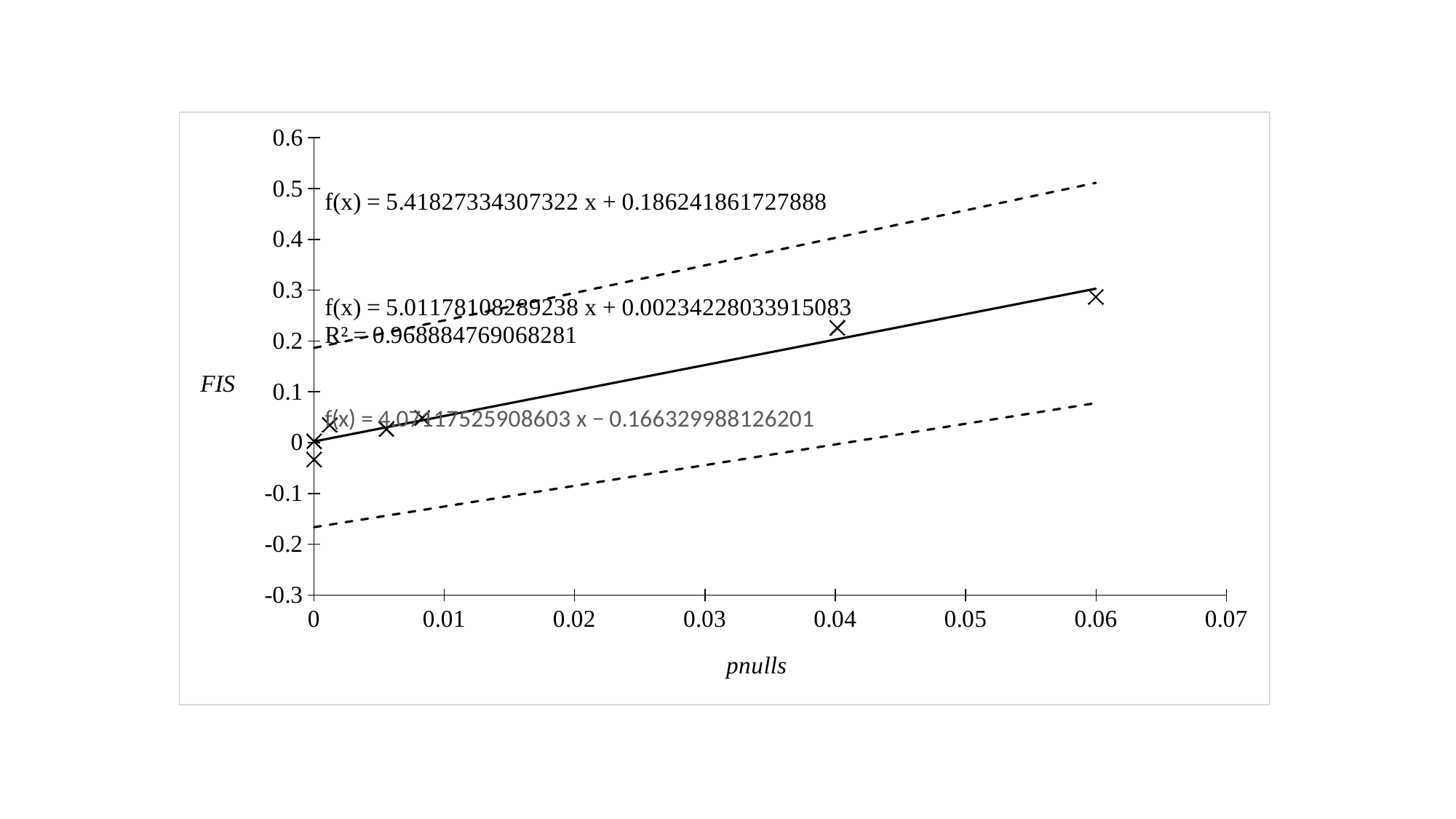

### Chart
| Category | FIS | FIS_i_B | FIS_s_B |
|---|---|---|---|

Supplement: Supplementary file 1 — Supplementary material 1: Entomological database. Traps Nb: Number of each trap PGE: Peripheral Gardening Area TotalGloss_TxMonthYear: Total number of tsetse flies captured per period of entomological monitoring (Tx) per month and per year ADT_TxMonthYear: Apparent Density per Trap per day per period of entomological monitoring (Tx) per month and per year Infect_T0MonthYear: Number of infected tsetse flies per period of entomological monitoring (Tx) per month and per year NS: Not Set T0: Trap set on sites identified during the January 2020 initial entomological survey Supplementary material 2: Genotyping data base FlyNb: Number of each tsetse fly Tx: Entomological monitoring XpGp13 to GPCAG: microsatellite loci Supplementary material 3: FIS results estimated within traps, zones or cohorts FIS are shown for each locus. The average FIS for all loci is represented by empty black circles. Supplementary material 4: Comparison of FIS between neutral loci FIS values are represented by solid black circles, and their 95% confidence intervals by dashes (bootstrap on individuals for each locus or on loci (All)). Significant deviations from panmictic expectations, the number of observed and expected missing data (in brackets) and the results of the stuttering test are also provided. Supplementary material 5: FIS regression as a function of the number of missing genotypes Regression of FIS against the number of missing genotypes with neutral loci only is represented by the single line with 95% CI bootstraps on individuals (FIS_i et FIS_s, dashed lines). All equations are given and the coefficient of determination (R2) is provided for the mean. Results for the XB110, XB104 and pGp24 loci are represented by an empty circle, a full circle and an empty square, respectively (black dashes represent 95% CIs). They do not participate in the regression. The other loci are represented by crosses. Supplementary material 6: Regression of FIS as a function of null allele frequencies (pnulls) [file parasite-32-25-s1.zip › parasite240104-1-olm/Suppl_mat_6_Fis_pnull.pptx]
